# Supplementary material for: Identification and functional analysis of non-coding regulatory small RNA FenSr3 in Bacillus amyloliquefaciens LPB-18
Source: PeerJ. 2023 May 15;11:e15236. doi: 10.7717/peerj.15236 (PMC10194069; doi:10.7717/peerj.15236)
Supplement: Supplemental Information 4 [file peerj-11-15236-s004.zip › KO/CK-vs-T1_map/map05214.html]

KEGG PATHWAY: Glioma - Reference pathway


|  |  |
| --- | --- |
| **Glioma - Reference pathway** |  |

[
Pathway menu
| Organism menu
| Pathway entry
| Show description
| User data mapping
]

Gliomas are the most common of the primary brain tumors and account for more than 40% of all central nervous system neoplasms. Gliomas include tumours that are composed predominantly of astrocytes (astrocytomas), oligodendrocytes (oligodendrogliomas), mixtures of various glial cells (for example,oligoastrocytomas) and ependymal cells (ependymomas). The most malignant form of infiltrating astrocytoma - glioblastoma multiforme (GBM) - is one of the most aggressive human cancers. GBM may develop de novo (primary glioblastoma) or by progression from low-grade or anaplastic astrocytoma (secondary glioblastoma). Primary glioblastomas develop in older patients and typically show genetic alterations (EGFR amplification, p16/INK4a deletion, and PTEN mutations) at frequencies of 24-34%. Secondary glioblastomas develop in younger patients and frequently show overexpression of PDGF and CDK4 as well as p53 mutations (65%) and loss of Rb playing major roles in such transformations. Loss of PTEN has been implicated in both pathways, although it is much more common in the pathogenesis of primary GBM.

|  |  |  |
| --- | --- | --- |
| Reference pathway | 184% 150% 122% 100% 82% 67% 55% | 图片下载 |
